# Ultrastructural Analysis of Cells From Bell Pepper (*Capsicum annuum*) Infected With Bell Pepper Endornavirus

**DOI:** 10.3389/fpls.2020.00491

**Published:** 2020-04-28

**Authors:** Katarzyna Otulak-Kozieł, Edmund Kozieł, Cesar Escalante, Rodrigo A. Valverde

**Affiliations:** ^1^Institute of Biology, Department of Botany, Warsaw University of Life Sciences—SGGW, Warsaw, Poland; ^2^Department of Plant Pathology and Crop Physiology, Louisiana State University Agricultural Center, Baton Rouge, LA, United States

**Keywords:** plant virus, *Endornaviridae*, electron microscopy, symptomless plants, plant organelle alterations, near isogenic lines, persistent virus

## Abstract

Endornaviruses include viruses that infect fungi, oomycetes, and plants. The genome of plant endornaviruses consists of linear ssRNA ranging in size from approximately 13-18 kb and lacking capsid protein and cell-to-cell movement capability. Although, plant endornaviruses have not been shown to cause detectable changes in the plant phenotype, they have been associated with alterations of the host physiology. Except for the association of cytoplasmic vesicles with infections by *Vicia faba* endornavirus, effects on the plant cell ultrastructure caused by endornaviruses have not been reported. Bell pepper endornavirus (BPEV) has been identified in several pepper (*Capsicum* spp.) species. We conducted ultrastructural analyses of cells from two near-isogenic lines of the bell pepper (*C. annuum*) cv. Marengo, one infected with BPEV and the other BPEV-free, and found cellular alterations associated with BPEV-infections. Some cells of plants infected with BPEV exhibited alterations of organelles and other cell components. Affected cells were located mainly in the mesophyll and phloem tissues. Altered organelles included mitochondrion, chloroplast, and nucleus. The mitochondria from BPEV-infected plants exhibited low number of cristae and electron-lucent regions. Chloroplasts contained plastoglobules and small vesicles in the surrounding cytoplasm. Translucent regions in thylakoids were observed, as well as hypertrophy of the chloroplast structure. Many membranous vesicles were observed in the stroma along the envelope. The nuclei revealed a dilation of the nuclear envelope with vesicles and perinuclear areas. The organelle changes were accompanied by membranous structure rearrangements, such as paramural bodies and multivesicular bodies. These alterations were not observed in cells from plants of the BPEV-free line. Overall, the observed ultrastructural cell alterations associated with BPEV are similar to those caused by plant viruses and viroids and suggest some degree of parasitic interaction between BPEV and the plant host.

## Introduction

Peppers (Capsicum species in the family Solanaceae) are native plants from the Americas and are cultivated worldwide as food crops (DeWitt and Bosland, 1996; Pickersgill, 1997). Although there are five domesticated Capsicum species (*C. annuum, C. baccatum, C. chinense, C. frutescens*, and *C. pubescens*), *C*. *annuum* is the most commonly cultivated (Bosland et al., 1996; DeWitt and Bosland, 1996; Pickersgill, 1997). Several *C. annuum* horticultural types have been identified, including bell, cayenne, jalapeño, ancho, serrano, poblano, and others (Smith et al., 1987).

Based on host symptom expression, plant viruses can be divided into two categories: acute and persistent (Roossinck, 2010). Acute viruses are transmitted horizontally and, in some cases, vertically. Their genome encodes for a cell-to-cell movement protein (MP), which in combination with other proteins gives them the ability to spread from the point of initial infection (Rojas et al., 2016). In contrast, persistent viruses do not cause morphological symptoms; they lack MP and are transmitted only vertically via gametes (Roossinck, 2010; Fukuhara, 2019). Persistent plant viruses include members of the families *Amalgaviridae, Chrysoviridae, Endornaviridae, Nardaviridae, Partitiviridae*, and *Totiviridae* (Roossinck, 2010; Nibert et al., 2018; Fukuhara, 2019; Takahashi et al., 2019). Persistent viruses have been reported to infect economically important crops such as alfalfa, avocado, corn, sugar beet, common bean, rice, pepper, melon, radish, and tomato (Boccardo et al., 1987; Fukuhara et al., 1993; Pfeiffer, 1998; Okada et al., 2011; Villanueva et al., 2012; Li et al., 2013; Sabanadzovic et al., 2016; Akinyemi et al., 2018). However, due to the lack of symptom induction and the lack of transmission by conventional methods, persistent viruses have been poorly studied. Interactions of persistent viruses with the host, acute viruses, and other biotic and abiotic agents have not been investigated.

Viruses in the family *Endornaviridae* infect fungi, oomycetes, and plants (Fukuhara, 2019; Valverde et al., 2019). The genome of plant endornaviruses consist of linear positive sense ssRNA ranging in size from approximately 13–18 k and lacking capsid protein (CP) and MP (Roossinck et al., 2011; Dolja and Koonin, 2018; Valverde et al., 2019). Indirect evidence suggests they are present in all tissues of the infected plant (Valverde et al., 2019). Like other persistent plant viruses, they have not been shown to cause visible phenotypic changes in the host (Khankhum and Valverde, 2018; Escalante and Valverde, 2019; Fukuhara, 2019). Nevertheless, plant endornaviruses have been associated with alterations of the host physiology such as seed germination, cytoplasmic male sterility, and chlorophyll content (Grill and Garger, 1981; Khankhum and Valverde, 2018; Escalante and Valverde, 2019).

Bell pepper endornavirus (BPEV) has been identified in many *C*. *annum* cultivars but particularly in the bell pepper horticultural type (Valverde et al., 1990; Okada et al., 2011; Safari and Roossinck, 2018). Moreover, a closely related virus, Capsicum frutescens endornaviurus 1 (CFEV 1), has been reported infecting several domesticated *Capsicum* species (Safari and Roossinck, 2018). Safari and Roossinck showed that BPEV occur only in *C. annuum*. In contrast, CFEV 1 was detected in *C. frutescens, C. chinense, and C. baccatum*. These results suggest that endornaviruses of *Capsicum* are not species-specific.

In a comparative study using near-isogenic lines (NILs) of bell pepper cv. Marengo, one BPEV-infected and the other BPEV-free, Escalante and Valverde (2019) determined that BPEV was not associated with changes in the host phenotype. However, the plant height, number of fruits, and total fruit weight was higher in plants of the BPEV-free line than in plants of the BPEV-infected line. However, in most experiments, the differences were not statistically significant. Escalante and Valverde (2019) concluded that BPEV appears to have a weak parasitic relationship with the host.

Except for the association of cytoplasmic vesicles in *Vicia faba* with infections by Vicia faba endornavirus (VfEV) (Dulieu et al., 1988), studies on the effects on the plant cell ultrastructure by endornaviruses have not been reported. One factor contributing to the lack of studies is that plant endornaviruses are not transmitted by conventional virus-inoculation methods; therefore, results from comparative studies using different plant genotypes are not reliable. The availability of BPEV-infected and BPEV-free near-isogenic lines provided us with material to conduct a comparative study to determine if ultrastructural changes in bell pepper are associated with BPEV infections. In this investigation, we conducted an ultrastructural analysis of leaf tissues of two near-isogenic lines of the bell pepper cultivar Marengo, one infected with BPEV and the other BPEV-free, and report the association of ultrastructural cytopathology with BPEV infections.

## Materials and Methods

### Plant Material

Seeds from two NILs of *C*. *annuum* cv. Marengo, one infected with BPEV and the other BPEV-free, developed in previous investigations (Escalante and Valverde, 2019) were planted and grown in a phytotron growth chamber at 20°C and 16 h light with an intensity of 400 μmol m-2 s-1 PAR (photosynthetically active radiation). The plant phenotype of both lines was visually examined (daily) throughout their life cycle. The presence or absence of BPEV in experimental plants was tested by analysis of viral replicative form dsRNA by gel electrophoresis and reverse transcription PCR (RT-PCR) as described in previous investigations (Okada et al., 2011; Khankhum et al., 2017). Furthermore, plants were tested for the presence of pepper mild mottle virus by RT-PCR (Jarret et al., 2008). Total RNA extracted from healthy tobacco (*Nicotiana tabaccum*) plants with the Spectrum Plant Total RNA Kit (Sigma-Aldrich, St. Louis, MO) was used as negative control in RT-PCR reactions.

### Tissue Preparation for Light Microscopy and Transmission Electron Microscopy (TEM) Examinations

Leaves from two-month-old plants of both NILs at similar developmental stage were selected for transmission electron microscope examinations. Thirty-five sections (2 mm^2^) were excised from each NIL and fixed as reported previously (Otulak-Kozieł et al., 2018, 2019). Briefly, tissues were initially fixed as described by Karnovsky (1965) and in 2% (w/v) osmium tetroxide solution in 0.05 M cacodylate buffer for 2 h at 4°C. Samples were dehydrated in ethanol series and embedded in Epoxy resin (Epon812, Sigma) with polymerization for 24 h at 60°C. For the examination of the anatomy, glass slides with macro-sections were stained with crystal violet solution (Otulak and Garbaczewska, 2010) and examined with a AX70 PROVIS light microscope with an Olympus UP90 High Definition camera (Olympus, Warsaw, Poland) using Olympus Cell Sense Standard Software (Olympus, Center Valley, PA, United States, version 1.18). Ultrathin sections (70–80 nm) were obtained using an UCT ultramicrotome (Leica Microsystems) and collected on formvar-coated copper grids. Grids were stained with 1.2% uranyl acetate and 2.5% lead citrate. Analyses of ultrathin sections from leaves of both NILs were performed using a transmission electron microscope (268D Morgagni TEM (FEI) at 80 kV) as previously described (Otulak-Kozieł et al., 2018, 2019). Images were captured with a Morada digital camera (Olympus SIS). All foliar sections were examined without knowledge of whether they were BPEV-infected or BPEV-free.

## Results

Gel electrophoresis and RT-PCR testing of plants of the two lines used for the ultrastructural analysis confirmed the presence of BPEV in BPEV-infected plants and absence of BPEV in BPEV-free plants (Supplementary Figures S1A,B). Moreover, pepper mild mottle virus was not detected in any of the experimental plants.

### Morphology and Anatomy of Near-Isogenic Lines (NILs)

**FIGURE 1:**
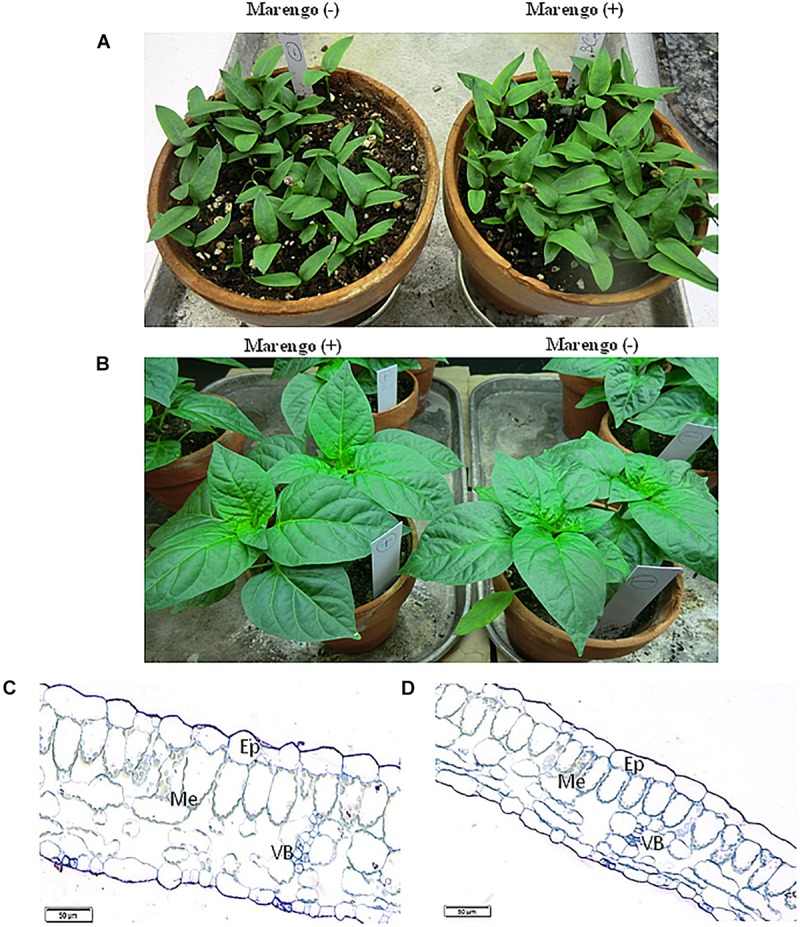
Morphology and anatomy of two near-isogenic lines of bell pepper cv. Marengo, one infected with BPEV [Marengo (+)] and the other BPEV-free [Marengo (−)]. **(A)** Seedlings, **(B)** Two- month-old plants. **(C,D)** Light microscopy of cross sections of tissues from two-month-old plants showing epidermis (Ep), Mesophyll (Me), and vascular bundles (VB). Bar 50 μm.

As reported in previous investigations (Okada et al., 2011; Escalante and Valverde, 2019), we did not observe phenotypical differences between the BPEV-infected and the BPEV-free bell pepper NILs (Figures 1A,B). Moreover, light microscopy examinations of cross sections of foliar tissues from both lines did not show visible differences on their anatomy (Figures 1C,D).

### Cellular Ultrastructural Changes Associated With Bell Pepper Endornavirus Infection

Electron microscopy examinations of foliar tissues of the two lines did not yield any evidence of virus-like particles or viral inclusion bodies.

Ultrastructural alterations of cell organelles and other components were observed in bell pepper tissues infected with BPEV. Some or all of these alterations were consistently observed in some cells of all 35 analyzed leaf sections. The altered cells were located mainly in the mesophyll and phloem tissues.

**FIGURE 2:**
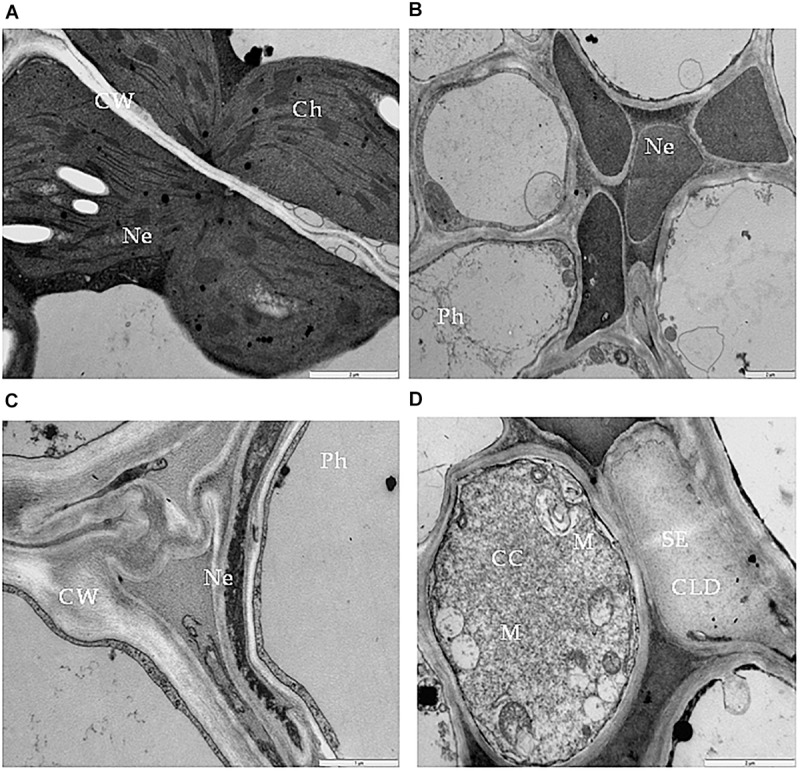
Necrosis in bell pepper tissues infected with BPEV. **(A)** Necrosis (Ne) of palisade mesophyll cells. Bar 2 μm. **(B)** Necrosis (Ne) in phloem (Ph) elements. Bar 2 μm. **(C)** Collapsed sieve element and necrosis of companion cell (CC). Bar 2 μm. **(D)** Sieve element (SE) filled with callose like deposition and companion cell (CC) with expanded mitochondria (M). Bar 2 μm.

**FIGURE 3:**
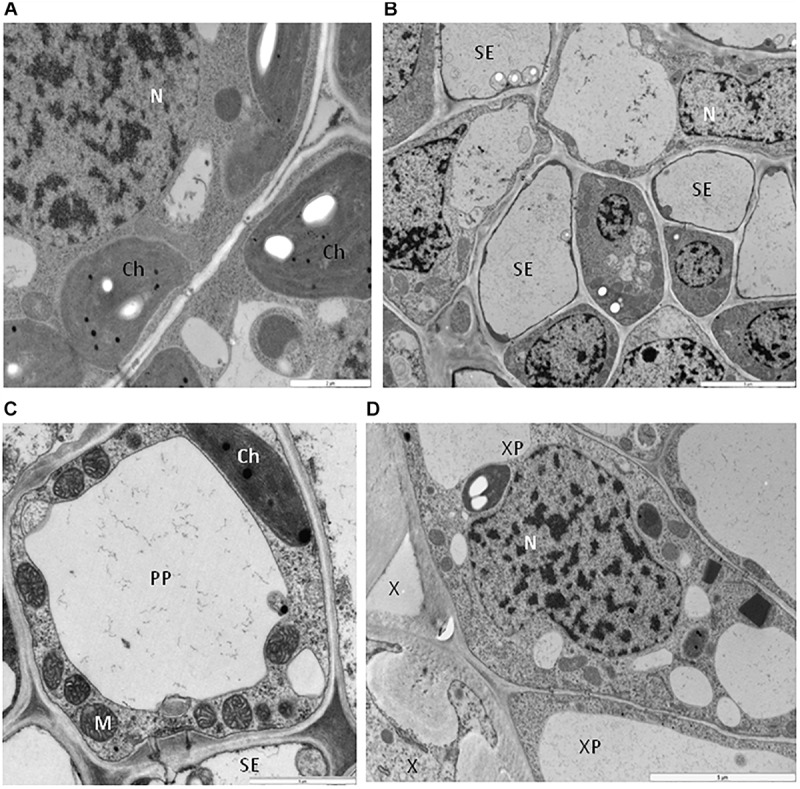
Cells of bell pepper tissues free of BPEV. **(A)** Spongy mesophyll cells. Ch-chloroplast, N-nucleus. Bar 2 μm. **(B)** Phloem cells. N-nucleus, SE-sieve element. Bar 5 μm. **(C)** Sieve element (SE) with companion cell (CC). Ch-chloroplast, M-mitochondria. Bar 5 μm. **(D)** Xylem cells. N-nucleus, X-xylem tracheary elements, XP-xylem parenchyma. Bar 5 μm.

**FIGURE 4:**
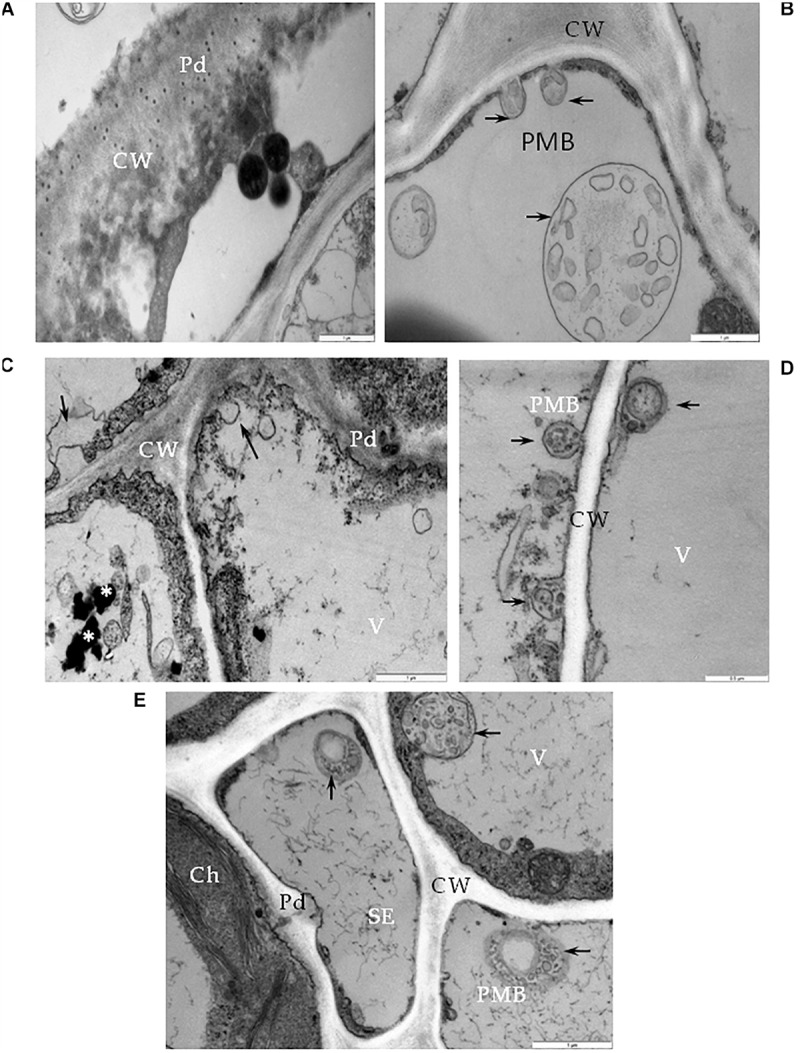
Cell wall alterations in cells of BPEV-infected tissues. **(A)** Phloem parenchyma (PP) cell wall (CW) loosening in the area near the plasmodesmata (Pd). Bar 1 μm. **(B)** Paramular bodies (PMB) formation (arrows) in epidermis cell. CW-cell wall. Bar 1 μm. **(C)** Changed cell wall (CW) structure with the formation of paramular bodies (arrows) in phloem parenchyma cells. Vesicular structures around phenolic compounds in vacuole (*). Pd-plasmodesmata. Bar 1 μm. **(D)** Paramular bodies (arrows, PMB) between cell wall (CW) and vacuole (V) in mesophyll cells. Bar 0.5 μm. **(E)** Paramular bodies (arrows, PMB) in sieve elements (SE) and companion phloem cells. Ch-chloroplast, Pd-plasmodesmata. Bar 1 μm.

**FIGURE 5:**
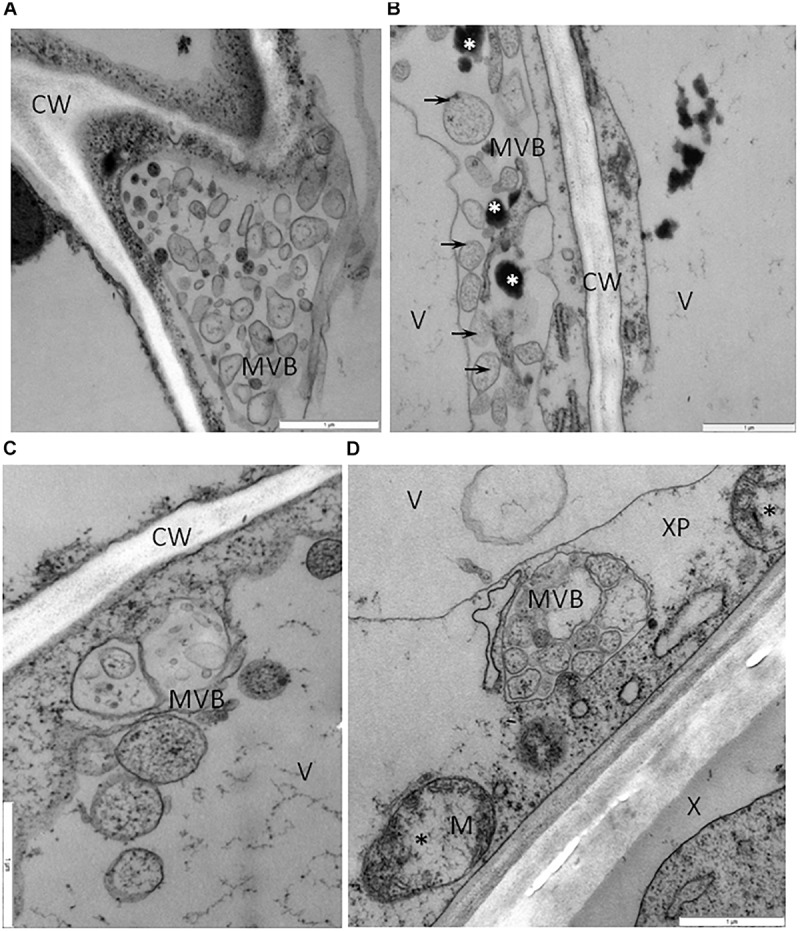
Vesicles and multivesicular structures in cells of BPEV-infected tissues. **(A)** Multivesicular structures (MVB) in vacuole of phloem parenchyma cell. CW-cell wall. Bar 1 μm. **(B)** Multivesicular structures (arrows, MVB) and phenolic compounds (white *) in cytoplasm of mesophyll cell. CW-cell wall, V-vacuole. Bar 1 μm. **(C)** Vesicles and multivesicular structures (MVB) in vacuole (V) of mesophyll cell. CW-cell wall bar 1 μm. **(D)** Multivesicular structures (MVB) in cytoplasm of xylem parenchyma (XP) cell. Mitochondria (M) with electron-lucent area (black *). X-xylem tracheary elements. Bar 1 μm.

One type of alteration consisted of necrosis of palisade mesophyll cells, which contained electron-dense cytoplasm (Figure 2A). Necrosis of some phloem elements was also observed (Figure 2B). The necrosis of the phloem cells was associated with collapsing of the sieve tubes and cell wall invaginations (Figure 2C). The altered ultrastructure of the sieve tubes was associated with abnormal companion cells. When compared with similar cells of the BPEV-free line (Figure 3), cells of the BPEV-infected line showed a decreased number of mitochondria and contained some mitochondria without crista (Figure 2D). In addition, these cells exhibited electron-dense regions along the cell wall, whereas, sieve tubes were filled with callose-like material (Figure 2D). Further analyses of phloem tissue cells revealed alterations of the cell wall. The spectrum of cell wall changes ranged from loss of cell wall structure, often near plasmodesmata (Figure 4A) to irregular cell wall invaginations associated with membranous paramural bodies (Figures 4B–E). Paramural bodies were observed associated with the cell wall of some phloem parenchyma, epidermis, and mesophyll cells. These paramular bodies were often located near the tonoplast of vacuoles, suggesting movement from the apoplast to the vacuoles. Membrane bound structures associated with the symplast region, such as multivesicular bodies were commonly observed in some cells from tissues of the BPEV-infected line. These multivesicular bodies varied in shape and occurred in the cytoplasm and vacuoles of mesophyll, phloem, and sometimes xylem parenchyma cells (Figures 5A–D). Numerous granular structures were frequently present inside the multivesicular bodies.

**FIGURE 6:**
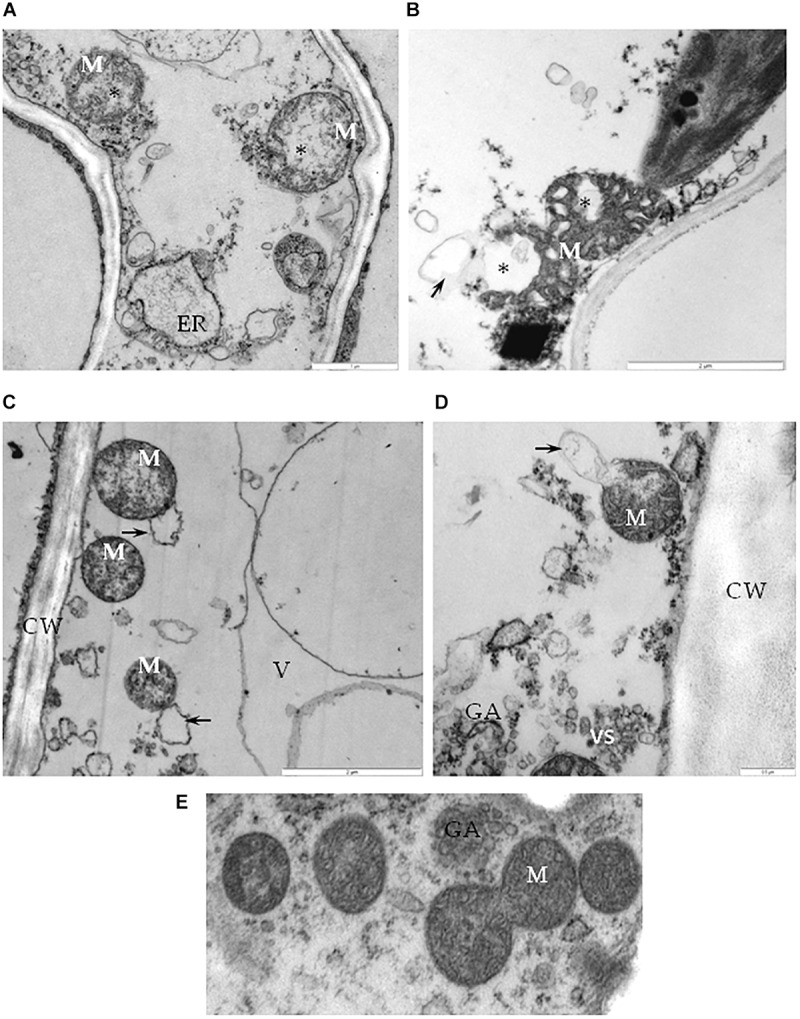
Alterations of mitochondria in cells of BPEV-infected tissues. **(A)** Mitochondria (M) with electron-lucent area (*) and expanded endoplasmatic reticulum (ER) in phloem parenchyma cell. Bar 1 μm. **(B)** Mitochondria (M) with an electron-lucent area (*) forming a vesicle-like structure (arrow) in mesophyll cell. Bar 2 μm. **(C)** Mitochondria (M) forming exosome like vesicles (arrows) in phloem parenchyma cell. CW-cell wall, V- vacuole. Bar 2 μm. **(D)** Mitochondria (M) with exosome like structure (arrow) in phloem companion cell. GA- trans Golgi network, vs-vesicles. Bar 0.5 μm. **(E)** Mitochondria (M) of a BPEV-free mesophyll cell. GA- trans Golgi network Bar 0.5 μm.

Further analysis of cells from the BPEV-infected line revealed ultrastructural changes in some cell organelles. The mitochondria exhibited a variety of structural alterations, which included a decrease number of crista and electron-lucent regions (Figures 6A,B). Mitochondria with electron-lucent areas formed an expanded exosome like vesicles (Figures 6C,D). None of these changes were observed in mitochondria of cells of the BPEV-free line (Figure 6E).

**FIGURE 7:**
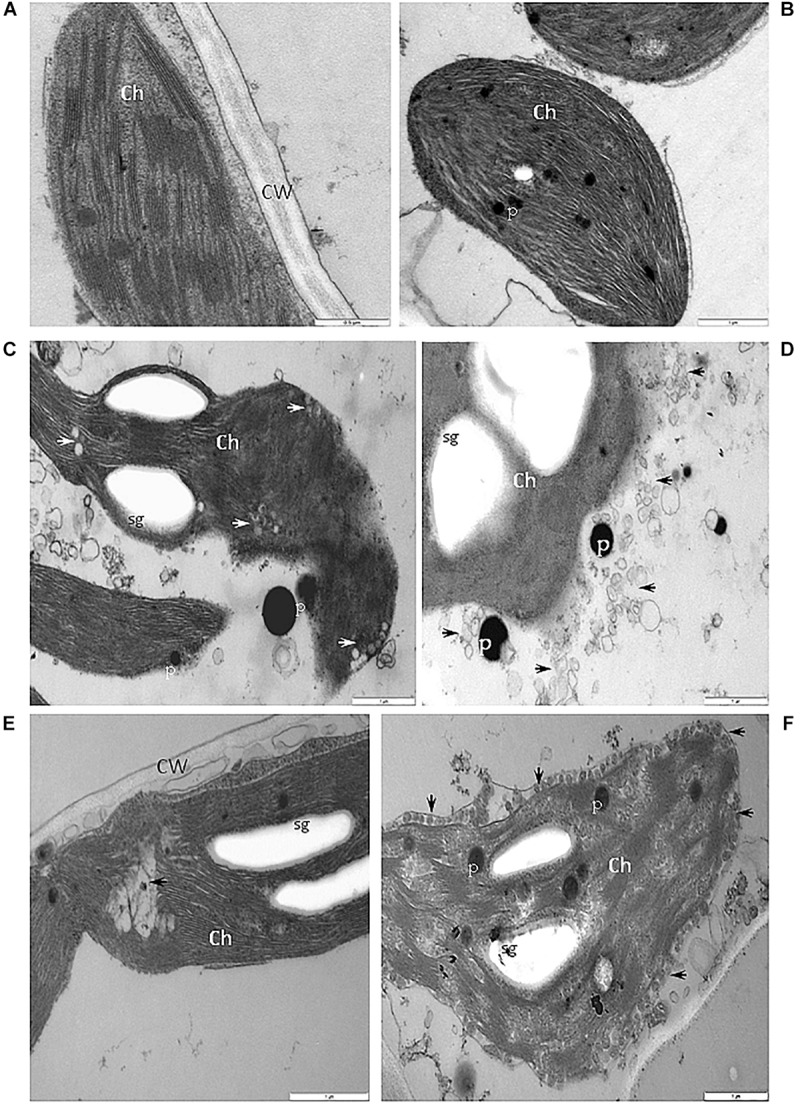
Alterations of the structure of chloroplasts in cells of BPEV-infected tissues. **(A)** Chloroplast (Ch) of a BPEV-free mesophyll cell. CW-cell wall. Bar 0.5 μm. **(B)** Abnormal structure of thylakoids. Ch-chloroplast, P-plastoglobules. Bar 1 μm. **(C)** Alteration of the chloroplast (Ch) morphology and small vesicles inside stroma (arrows). P-plastoglobules, sg-starch grains. Bar 1 μm. **(D)** Small vesicles (arrows) and plastoglobules (P) between the chloroplast (Ch) and the chloroplast membrane. sg-starch grains. Bar 1 μm. **(E)** Electron lucent area (arrow) inside chloroplast (Ch). CW-cell wall, sg-starch grains. Bar 1 μm. **(F)** Small vesicles (arrows) of stroma fragmentation and altered chloroplast (Ch) envelope. P-plastoglobules, sg-starch grains. Bar 1 μm.

When compared with chloroplast from cells of the BPEV-free line (Figure 7A), alterations of the chloroplast structure was observed in cells of the BPEV-infected line. The structure of the chloroplast thylakoids was altered (Figure 7B). Changes of the normal chloroplast shape and presence of small vesicles inside stroma were observed in some BPEV-infected cells (Figure 7C). Plastoglobules and small vesicles were often observed outside the chloroplast envelope (Figures 7C,D). Hypertrophy of the chloroplast structure included translucent regions in thylakoids and the presence of numerous membranous vesicles in stroma along envelope (Figures 7E,F).

**FIGURE 8:**
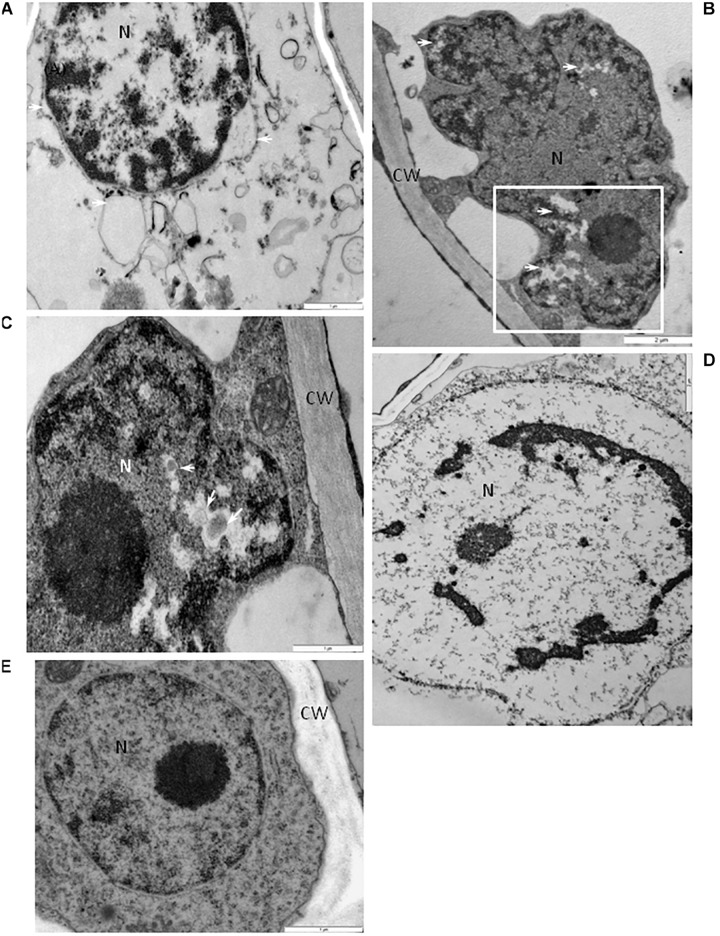
Alterations of the nuclear structure in cells of BPEV-infected tissues. **(A)** Dilatation of the nuclear envelope (arrows) with vesicles in the perinuclear areas. N-nucleus. Bar 1 μm. **(B)** Nucleus (N) with electron-lucent areas (arrows) in a mesophyll cell. CW-cell wall. Framed area enlarged in **(C)**. Bar 2 μm. **(C)** Enlargement from the framed area of figure **(B)**. Small vesicles (arrows) in electron-lucent area. CW-cell wall, N-nucleus. Bar 1 μm. **(D)** Chromatin condensation in nucleus (N). Bar 1 μm. **(E)** Nucleus (N) of a BPEV-free phloem parenchyma cell. CW-cell wall. Bar 1 μm. CC, companion cell; Ch, chloroplast; CLD, callose like deposition; CW, cell wall; ER, endoplasmic reticulum; Ep, epidermis; GA, trans Golgi network; M, mitochondria; Me, mesophyll; MVB, multivesicular bodies; N, nucleus; Ne, necrosis; P, plastoglobules; Ph, phloem; PMB, paramular bodies; SE, sieve element; sg, starch grain; vs, vesicles; V, vacuole; VB, vascular bundle; X, xylem tracheary element; XP, xylem parenchyma.

Some phloem parenchyma cells from the BPEV-infected line contained nuclei with dilation of the nuclear envelope and vesicles and perinuclear areas (Figure 8A). Whereas, in the mesophyll cells, lobed nucleus with translucent regions containing small vesicles were often observed (Figures 8B,C), including strong chromatin condensation (Figure 8D). None of the changes in cell organelles and other cell components described above were observed in cells of BPEV-free line (Figure 8E).

The alterations described above were not observed in all cells of the BPEV-infected line. Many cells of the BPEV-infected line contained unaltered organelles and other cell components which were undistinguishable from the organelles and cell components of the BPEV-free line.

## Discussion

Previous studies have reported that plants infected with endornaviruses are symptomless (Khankhum and Valverde, 2018; Escalante and Valverde, 2019; Fukuhara, 2019). The genomes of these viruses do not code for a CP, and therefore, it is not surprising that virions have not been reported in endornavirus-infected cells (Valverde et al., 1990; Zabalgogeazcoa and Gildow, 1992; Fukuhara, 1999). Limited studies on the cellular location of the RNA of plant endornaviruses suggest that they are concentrated in the cytoplasm (Lefebvre et al., 1990; Valverde et al., 1990; Okada et al., 2013; Liu et al., 2018). The replicative form of the genomic RNA (dsRNA) of VfEV-infected *V. faba* has been found to be associated with cytoplasmic vesicles and viral dsRNA isolated from purified vescicles (Lefebvre et al., 1990). As mentioned earlier in this paper, at the present time, other than the vesicles in VfEV-infected cells, no cytopathic effects have been reported as associated with endornaviruses, and there is no information on the effect of endornaviruses to cell organelles or other cell components.

The formation of cytoplasmic vesicles is one of the most common cellular responses to infection of plants by viruses (Francki, 1987). In this investigation, we observed cytoplasmic vesicles and multivesicular bodies in BPEV-infected cells which were similar to those reported in plants infected with acute viruses. In plant cells, single-stranded positive-sense RNA viruses generate cytoplasmic membranous vesicles, where viral replication takes place (Wei and Wang, 2008). These vesicles contain viral ssRNA, replicative dsRNA, and proteins involved in virus replication (Lefebvre et al., 1990; Cotton et al., 2009; Cabanillas et al., 2018). Vesicles can be developed from membranes of various cell organelles such as chloroplasts, mitochondria, peroxisomes, endoplasmic reticulum, or tonoplast (Hatta and Ushiyama, 1973; Hatta and Francki, 1980; Martelli et al., 1984; Laliberté and Zheng, 2014). Multivesicular bodies have been reported in cells infected with tomato bunchy top virus (Martelli et al., 1984). Cytoplasmic vesicles apparently formed by invaginations of the plasma membrane have been reported in tomato plants infected with potato spindle tuber viroid. Membrane-bound vesicles (50-90 nm in diameter) have been observed in fungi infected with hypoviruses (Newhouse et al., 1990; Khalifa and Pearson, 2014). Moreover, the presence of a large number multivesicular bodies has been associated with viral genome replication (Laliberté and Sanfaçon, 2010; Laliberté and Zheng, 2014). Multivesicular bodies have also been associated with cell wall-associated defense response in barley leaves infected with the pathogen that causes powdery mildew (An et al., 2006a, b). Cytoplasmic vesicles and multivesicular bodies generated in BPEV-infected cells suggest involvement of these structures in the virus replication as reported for brome mosaic virus (Bamunusinghe et al., 2011). Paramural bodies observed in cells infected with BPEV are similar to paramular bodies observed in the potato virus Y (PVY)-resistant potato cultivar Sárpo Mira when infected with PVY (Otulak-Kozieł et al., 2018). Paramural bodies and membrane alterations have also been reported in cells infected with potato spindle tuber viroid (Hari, 1980).

Plant viruses have been shown to target photosynthesis and negatively affect the chloroplast function, including host chlorophyll content (Zhao et al., 2016). Morphological changes of the chloroplast have been reported to be associated with plant virus or viroid infections (Hari, 1980; Lin and Langenberg, 1984). It has been shown that the chloroplasts of potato spindle tuber viroid-infected cells exhibit reduced grana and loosely arranged thylakoids (Hari, 1980). Lin and Langenberg (1984) reported that barley stripe mosaic virus caused alterations of the wheat chloroplast membranes, characterized by the clustering of outer membrane-invaginated spherules in inner membrane-derived packets. They also observed diverse morphologies of cytoplasmic invaginations with spherules at the periphery and different sized openings connecting the cytoplasmic invaginations with the cytoplasm (Lin and Langenberg, 1984). We observed similar changes of the chloroplast and cytoplasm in cells of the BPEV-infected line. In a comparative study of endornavirus-infected and endornavirus-free common bean (*Phaseolus vulgaris)*, Khankhum and Valverde (2018) reported statistically significantly lower chlorophyll content of the endornavirus-infected line. Although in the case of bell pepper and BPEV, Escalante and Valverde (2019) did not find statistically significant differences on the amount of chlorophyll content between infected and healthy lines.

The mitochondria in BPEV-infected cells exhibited a variety of structural alterations which included electron-lucent areas and expanded exosome like vesicles. In turnip, infections by turnip mosaic virus causes vesicularization of the outer mitochondrial membranes (Blake et al., 2007; Otulak and Garbaczewska, 2013; Otulak et al., 2015). Electron microscopic observations of thin sections of cells from tissues infected with cucumber green mottle mosaic virus revealed the formation of small vesicles in the mitochondria (Hatta and Ushiyama, 1973). Gómez-Aix et al. (2015) reported that melon necrotic spot virus replication occurs in association with altered mitochondria. Cytoplasmic vesicles that develop from modified mitochondria have been shown to be associated with infections by tombusviruses (Di Franco et al., 1984). Park et al. (2006) reported smaller and fewer mitochondria in the fungus *Chalara elegans* infected with a mitovirus. Mitoviruses are small RNA viruses that infect plants and fungi and replicate in the mitochondria (Nibert et al., 2018).

Many of the cellular alterations reported in this investigation resemble effects caused by biotic and abiotic stresses in plants. Although infected bell pepper plants containing the described cell alterations did not appear diseased, these alterations should negatively affect the normal plant physiology. In spite of the presence of clusters of necrotic cells, we did not observe tissue necrosis. It is possible that the number of necrotic cells was below the threshold to cause visible necrosis. The observed cellular alterations may explain the lower seed germination, plant height, number of fruits, and total fruit weight of BPEV-infected plants than plants of the BPEV-free line reported by Escalante and Valverde (2019).

While conducting investigations on the coevolution of *Capsicum* endornaviruses and the host, Safari and Roossinck (2018) generated data to support the idea that the ancestor of CFEV 1, may have evolved as BPEV in *C. annuum*. In the United States, BPEV has been detected in all tested bell pepper (*C. annuum*) cultivars (Okada et al., 2011; Escalante and Valverde, 2019). This suggests that bell pepper breeders have selected only BPEV-infected lines to develop commercial cultivars and therefore BPEV may provide an unknown beneficial effect to the plant. Similarly, most melon cultivars tested for Cucumis melo endornavirus have been found infected (Sabanadzovic et al., 2016). Nevertheless, it is possible that the beneficial effects may be effective only under certain environmental conditions. Common bean (*Phaseolus vulgaris*) cultivars of Mesoamerican origin have been reported to be double-infected by two endornaviruses, Phaseolus vulgaris endornavirus 1 (PvEV1) and Phaseolus vulgaris endornavirus 2 (PvEV2), whereas most genotypes of Andean origin were endornavirus-free (Okada et al., 2013; Khankhum et al., 2015). This differential occurrence according to the crop origin suggests that like BPEV, PvEV1 and PvEV2 may provide unidentified beneficial effects to common bean grown in Mesoamerica but not to that grown in the Andean region. A differential infection pattern has also been reported for Oryza sativa endornavirus in Indica and Japonica rice (Fukuhara et al., 1993).

Only limited studies on the association of endornaviruses with changes in the host biology have been conducted (Grill and Garger, 1981; Khankhum and Valverde, 2018; Escalante and Valverde, 2019). Endornaviruses of common bean and bell pepper have been associated with statistically significant variations in seed germination rates (Khankhum and Valverde, 2018; Escalante and Valverde, 2019). There is evidence that suggests that endornaviruses activate the plant host gene silencing system and therefore play an active role in the physiology of the infected plant (Urayama et al., 2010; Sela et al., 2012). The lack of symptom induction suggests that endornaviruses are able to evade the silencing mechanism of the host, possibly by using a unique suppressor of silencing or other unknown mechanism. Fukuhara (2019) has suggested that the host regulates endornavirus copy number and propagation and that unknown host factors, which could be proteins involved in RNA silencing, control virus replication. Nevertheless, in the case of bell pepper, alterations of the ultrastructure of some host cells were associated with BPEV infections without causing visible external symptoms. Although not yet experimentally confirmed, it is assumed that endornaviruses are present in all cells of an infected plant; we do not have an explanation for the presence of cells with altered and non-altered organelles and other cell components in BPEV-infected plants. It is possible that alterations occur only in cells lacking host control on endornavirus replication.

## Data Availability Statement

All datasets generated for this study are included in the article/Supplementary Material.

## Author Contributions

KO-K and EK conducted the light and electron microscopy experiments, data analyses, and participated in writing the manuscript. CE conducted dsRNA extractions and RT-PCR testing. RV developed bell pepper near-isogenic lines and participated in writing the manuscript.

## Conflict of Interest

The authors declare that the research was conducted in the absence of any commercial or financial relationships that could be construed as a potential conflict of interest.
