# Supplementary figures and images for: Ultrastructural Analysis of Cells From Bell Pepper (Capsicum annuum) Infected With Bell Pepper Endornavirus
Source: Front Plant Sci. 2020 Apr 28;11:491. doi: 10.3389/fpls.2020.00491 (PMC7199235; doi:10.3389/fpls.2020.00491)

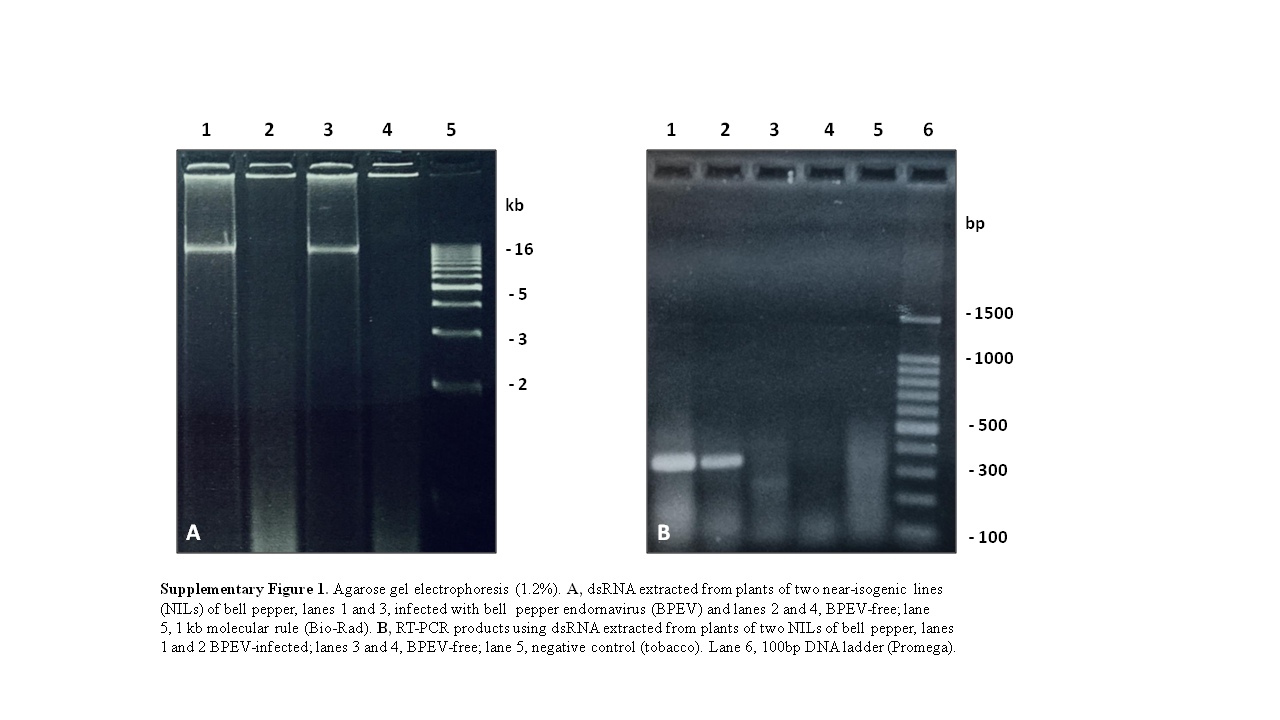

Supplement: Supplementary file 1 [file Image_1.tif]
